# Assessment of knowledge of human papillomavirus transmission and prevention among tertiary institution students in the Plateau Central Senatorial District, Nigeria

**DOI:** 10.1371/journal.pgph.0003273

**Published:** 2024-09-27

**Authors:** Juliana Rume, Imran O. Morhason-Bello, Adesina Oladokun

**Affiliations:** 1 Pan African University, Life and Earth Science Institute (including Health and Agriculture), University of Ibadan, Ibadan, Oyo State, Nigeria; 2 College of Education Gindiri, Gindiri, Plateau State, Nigeria; 3 Obstetrics and Gynaecology Department, Faculty of Clinical Sciences, College of Medicine/University College Hospital, University of Ibadan, Ibadan, Oyo State, Nigeria; 4 Institute of Advance Medical Research and Training, College of Medicine, University of Ibadan, Ibadan, Oyo State, Nigeria; 5 HPV Research Consortium, College of Medicine, University of Ibadan, Ibadan, Oyo State, Nigeria; University of Cape Town, SOUTH AFRICA

## Abstract

Human papillomavirus infection (HPV) is a significant global public health concern, known to be a leading cause of cancer related death among women in sub-Saharan Africa. This study assessed knowledge of HPV infection, transmission, prevention, and HPV vaccine uptake among tertiary institution students in Plateau State, Nigeria. A cross-sectional study was conducted among students from two tertiary institutions in Plateau State, Nigeria. Using a structured pretested questionnaire, data were collected from participants selected by two-stage sampling technique. Participants’ responses were analysed to assess their knowledge regarding HPV transmission, prevention, and vaccination. A composite score was obtained for the general HPV knowledge. A score of more than 70.0% indicated good knowledge. The distribution of the variables was examined using frequency distribution and descriptive statistics. The chi-square test was performed for bivariate analysis. Logistics regression was performed to examine the odds of having good HPV knowledge among the students. Level of significant was set at 95%. Of the 425 participants, 302 (71.1%) were female and 123 (28.9%) were male, with a mean age of 23 ± 2.8 years. There was low awareness of HPV among participants, with higher awareness among the females 66 (23.1%) than the males 26 (22.2%) (p = .853). Both female 77 (26.1%) and male participants 31 (26.72%) had low awareness of HPV vaccination. Among all participants, only 19 (5%) demonstrated good knowledge of HPV. Participants who were employed significantly had good knowledge of HPV compared to those who were unemployed. There was inadequate general knowledge of HPV and its vaccination among tertiary institution students in Plateau State, Nigeria. The students’ employment status was associated with their knowledge of HPV. Targeted educational programs focusing on diverse educational levels and institution types are recommended to enhance HPV knowledge and promote vaccine uptake.

## Introduction

Human papillomavirus (HPV) is a non-enveloped, double-stranded, circular DNA virus that is responsible for causing multiple epithelial lesions and cancers in both genders. It can manifest as cutaneous and anogenital warts, which depending on the subtype, may progress to carcinoma [1, 2]. HPV is predominantly transmitted through sexual contact, including vaginal, anal, and oral sex [3, 4]. Multiple health problems, such as genital warts, and cancers of the mouth/throat, cervix and anogenital sites are due to persistence of HPV infections [4]. Non-sexual skin contacts and vertical transmission from mothers to babies during childbirth are other known modes of transmission [4, 5]. Generally, HPV infections are classified into high or low risk HPV depending on their oncogenic potentials. Anogenital and cutaneous warts are caused by low-risk HPVs (LR-HPVs), while anogenital (cervical, anal, vulvar, vaginal, and penile) cancers and oropharyngeal (tongue, tonsil, and throat) cancers are caused by high-risk HPVs (HR-HPVs) [6, 7]. Most sexually active men and women will be infected with HPV at some point in their life, and some may get it more than once [8].

HPV infection constitutes a major global public health concern due to its high prevalence, especially in women, where it is the main cause of cancer. Cervical HPV infection and related cancers have a varying geographic burden; they are primarily observed in low- and middle-income South American, African, and Asian countries. Previous studies indicated that Africa had the highest cervical HPV prevalence (21.1%), followed by Europe (14.2%), America (11.5%), and Asia (9.4%) among women with normal cervical cytology (NCC) [9–11].

Nigeria is the most populous country in Sub-Saharan Africa with estimated population of over 200 million people [12]. Numerous studies have shown high prevalence of HPV ranging between 16.1% to 68.8% among different population in Nigeria [13–18]. This prevalence is higher among younger women, particularly those under 30 years of age, and gradually decreases as women age in Nigeria [13, 18]. Despite much research on HPV awareness and prevention in Nigeria, there is limited information about HPV knowledge among tertiary institution students in the Plateau Central Senatorial District, Nigeria, to the best of our knowledge. Public health programs to enhance young adults’ knowledge regarding HPV symptoms, causes, and prevention are also lacking. As a result, this study aimed to assess knowledge of the transmission and prevention of HPV and HPV vaccine uptake among students in tertiary institutions in Plateau State. This study is very important for public health in Plateau State, Nigeria, because it has potential to influence policy and programs among young people in Northcentral region and their associated socio-cultural peculiarities.

## Materials and methods

### Study design

This cross-sectional study was conducted among students in tertiary institutions in Plateau State to assess HPV awareness and knowledge on its transmission, prevention, and vaccination.

### Study setting

Data were collected from students at two different tertiary institutions in Plateau state: Plateau State University Bokkos (PLASU) and the Federal College of Education (FCE) Pankshin. Plateau State is one of the six states located in North Central Nigeria. It has a unique plateau landscape and a temperate climate. Plateau State has an estimated population of 3.5 million people, comprising over 50 ethnic groups [19]. The Plateau Central Senatorial District consists of five local governments, including Pankshin and Bokkos, each of which houses one of the institutions where data were collected for this study [19].

PLASU is a medium-sized public non-profit degree-awarding institution located in the small city of Bokkos, with a total student population of 7,000. It is officially recognized by the National Universities Commission of Nigeria and offers courses and programs leading to accredited higher education degrees in various fields of study [20]. FCE Pankshin is a teacher-training institution that awards both first degrees in education and the Nigerian Certificate of Education (NCE). It currently has 13,269 students enrolled in NCE and degree programs across seven schools: School of Education, School of Arts and Social Sciences, School of Languages, School of Vocational and Technical Education, among others. FCE Pankshin offers 78 course combinations, including eight double major courses, all accredited by the National Commission of Colleges of Education (NCCE). These institutions were purposively selected because their location is central on the Plateau, connecting the northern and southern parts of the state [21, 22].

### Inclusion criteria

Students who have studied for at least one semester at each of the selected institutions, are between 18 and 30 years old, and signed the consent form to participate in the study.

### Exclusion criteria

Among those who met the inclusion criteria, students were excluded if they were not available during data collection or did not fully participate in the study.

### Sampling techniques

A two-stage sample technique was used to select the study participants. In the first stage, PLASU and FCE Pankshin were purposively selected from the list of tertiary institutions in Plateau State. In the second stage, students that showed interest after seeing the poster displayed at the strategic locations or from online advertisement on student’s social media pages visited the data collection points were invited and recruited.

### Sample size

The required sample size was obtained using the cross-sectional study sample size formula:

$$n=\frac{{Z_{\frac{\alpha }{2}}}^{2}p(1-p)}{d^{2}}$$

Where:

n is the sample size

$Z_{\frac{\alpha }{2}}$ is the level of confidence (95%) = 1.96

p is the proportion of participants with awareness of HPV from the previous study = 56.4%

d is the precision of the study set at 5%.

The required sample size was calculated to be 379. To accommodate potential non-response biases, 12% was added to the estimated sample size, resulting in a total sample size of 425 used for the study. The 12% adjustment provided a reasonable buffer to account for potential non-responders without excessively inflating the sample size, while ensuring feasibility in terms of time, budget, and manpower for data collection.

### Participants recruitment and instrument for data collection

A structured questionnaire was developed for data collection following a review of the literature and discussions with Sexual Health Educators (S1 Text). After the design phase, another set of independent experts (a Public Health Epidemiologist and Obstetrician and Gynecologists) reviewed the questions for reliability before we conducted a pilot test. The questionnaire consisted of closed-ended questions to assess the participants’ awareness of HPV, knowledge of transmission, preventive practices, and vaccination status, and was administered in English. The reliability of the questionnaire in assessing HPV knowledge was determined using Cronbach’s alpha. The internal consistency was strong, with a Cronbach’s alpha value of 0.86. Before data collection, the questionnaire was pretested at another higher institution in Plateau State (College of Education Gindiri) to ensure clarity and appropriateness. Based on the results of the pilot test, the educational level was adjusted to accommodate students who are spending extra years on campus. The wording and tone of the questions were also edited to ensure greater clarity.

Participants were recruited through banners, student social media groups, and the students’ union government (SUG) officials. The questionnaires were distributed to consenting participants at the institutions. Data collection for the study was conducted at FCE Pankshin between May 4th and 6th, 2023, and at PLASU between May 22nd and 24th, 2023.

### Data management and analysis

Data collected were screened for completeness, coded, and analysed using STATA (StataCorp L.L.C.). Descriptive statistics and frequency distribution were obtained to assess the distribution of the variables. The chi-square statistical test was used to determine the association between the socio-demographic characteristics of the participants and their awareness and knowledge of HPV and its vaccination. Logistics regression was performed to examine the odds of having good HPV knowledge among the participants. The missing data were included in the descriptive analyses but were excluded from the tests of associations and multivariable models. For bivariate analysis, a composite score was computed. The assessment of HPV knowledge involved questions covering the mode of transmission of HPV, whether HPV can cause cervical cancer, and methods of HPV infections prevention. Each correct answer was assigned a score of 1, while an incorrect answer received a score of 0. The total score was converted to a percentage and classified as follows: a score of less than or equal to 70.0% indicated a poor level of knowledge, while a score higher than 70% indicated a good level of knowledge. A p-value less than 0.05 was considered statistically significant.

### Ethical consideration

Ethics Committee (EC) of the Institute for Advanced Medical Research and Training (IAMRAT), College of Medicine, University College Hospital, University of Ibadan, Nigeria, gave ethical approval for this work, with reference number UI/EC/22/0028. Administrative clearance was obtained from the Plateau State Ministry of Health and the Plateau State Ministry for Higher Education. Further permission was granted by FCE in Pankshin and PLASU in Bokkos, Plateau State, where the research data were collected. A written consent form was attached to the questionnaire which participants all read and those who were willing to participate signed prior to filling of the questionnaire. The informed consent contained the study objectives, study procedure, assurance of confidentially, the voluntary nature of their participation and also assurance that the data obtained will be anonymized to protect their privacy.

## Results

Among the 425 participants selected from the two tertiary institutions, 302 (71.1%) were female, and 123 (28.9%) were male. Participants from both institutions had an average age of 23 years. Of the total participants, 45 (10.6%) were married, while the majority, 380 (89.4%), were single. More than half, 289 (68.5%), of the participants were unemployed, while 133 (31.5%) reported being employed (**Table 1**).

**Table 1:** Demographic characteristics of respondents by institutions.

Variables	Total N = 425	PLASU N = 179	FCE N = 246
	n (column %)	n (column %)	n (column %)
Age^1^ [mean(sd)]	23.3 (2.8)	23.3 (3.1)	23.3 (2.5)
**Gender**			
Female	302 (71.1)	131 (73.2)	171 (69.5)
Male	123 (28.9)	48 (26.8)	75 (30.5)
**Educational Level** ^2^			
100L	110 (26.0)	83 (46.9)	27 (11.0)
200L	93 (22.0)	34 (19.2)	59 (24.0)
300L	176 (41.6)	25 (14.1)	151 (61.4)
400L	34 (8.0)	34 (19.2)	0
Others	10 (2.4)	1 (0.6)	9 (3.7)
**Marital Status**			
Married	45 (10.6)	9 (5.0)	36 (14.6)
Single	380 (89.4)	170 (95.0	210 (85.4)
**Religion** ^3^			
Christianity	406 (96.7)	174 (98.3)	232 (95.5)
Islam	14 (3.3)	3 (1.7)	11 (4.5)
**Employment** ^4^			
Yes	133 (31.5)	64 (36.2)	69 (28.2)
No	289 (68.5)	113 (63.8)	176 (71.8)

1–32 missing, 2–2 missing, 3–5 missing, 4–3 missing, Others—Students who are spending extra years on campus

Generally, there was low awareness of HPV among the participants, with only 92 (22.8%) indicating awareness. More female participants 66 (23.1%) were aware of HPV compared to male participants 26 (22.2%) (**Table 2**). University participants 47 (27.2%) showed higher awareness of HPV than College of Education participants 45 (19.6%). There was no discernible difference in HPV awareness between married and single participants; however, employed participants demonstrated more awareness of HPV than those without a job. Only 108 (26.3%) participants were aware of the HPV vaccine. Both female 77 (26.10%) and male 31 (26.72%) participants showed low awareness of HPV vaccination. Similar to HPV awareness, employed participants 41 (31.54%) were more aware of the HPV vaccine than those unemployed 66 (23.74%). However, College of Education participants 74 (31.49%) showed more awareness of the HPV vaccine than university participants 34 (19.32%). The type of institution (p = 0.006) was significantly associated with awareness of HPV vaccine (**Table 2**).

**Table 2:** Awareness of HPV and its vaccination by respondents’ demographic characteristics.

Variables	Awareness of HPV^1^		p-value	Awareness of HPV vaccination^2^		p-value
	Yes N = 92	No N = 311		Yes N = 108	No N = 303	
	n (column %)	n (column %)		n (column %)	n (column %)	
Age [mean(sd)]	23.8 (0.3)	23.1 (0.2)	.063	23.2 (0.2)	23.3 (0.2)	.681
**Gender**			.853			.897
Female	66 (23.1)	220 (76.9)		77 (26.1)	218 (73.9)	
Male	26 (22.2)	91 (77.8)		31 (26.7)	85 (73.3)	
**Institution**			.072			.006
PLASU	47 (27.2)	126 (72.8)		34 (19.3)	142 (80.7)	
FCE	45 (19.6)	185 (80.4)		74 (31.5)	161 (68.5)	
**Educational Level**			.050			.396
100L	26 (24.8)	79 (75.2)		24 (21.8)	86 (78.2)	
200L	19 (21.1)	71 (78.9)		28 (31.8)	60 (68.2)	
300L	32 (19.6)	131 (80.4)		48 (28.6)	120 (71.4)	
400L	14 (42.4)	19 (57.6)		6 (18.3)	27 (81.8)	
Others	1 (10.0)	9 (90.0)		2 (20.0)	8 (80.0)	
**Marital Status**			.986			.254
Married	10 (22.7)	34 (77.3)		15 (33.3)	30 (66.7)	
Single	82 (22.8)	277 (77.2)		93 (25.4)	273 (74.6)	
**Religion**			.221			.615
Christianity	86 (22.2)	301 (77.8)		103 (26.1)	291 (73.9)	
Islam	4 (36.4)	7 (63.6)		3 (25.0)	9 (75.0)	
**Employment**			.648			.095
Yes	31 (24.4)	96 (75.6)		41 (31.5)	89 (68.5)	
No	61 (22.3)	212 (77.7)		66 (23.7)	212 (76.3)	

1–22 missing, 2–14 missing, Others—Students who are spending extra years on campus

**Fig 1:**
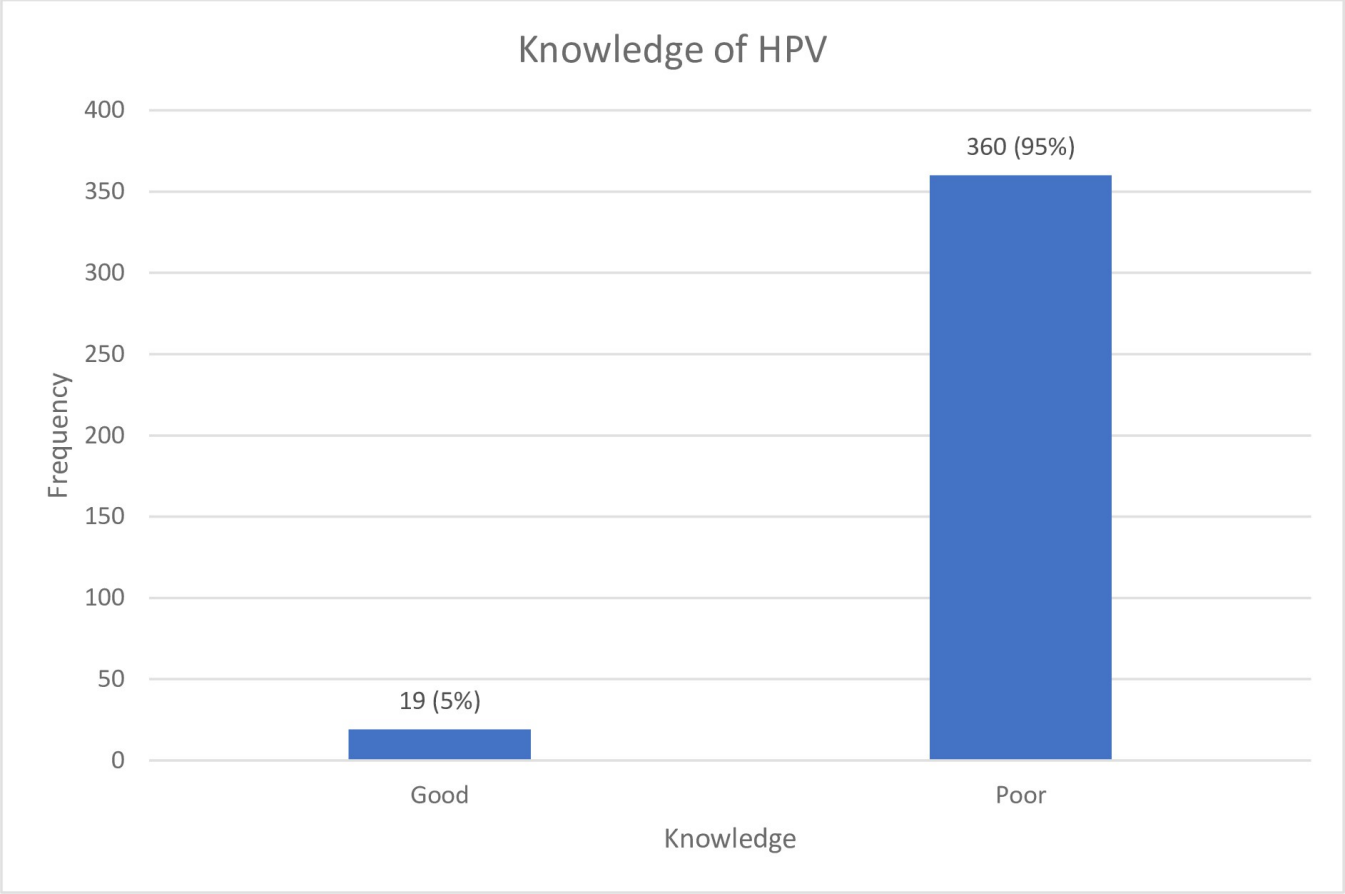
Students general knowledge of HPV.

The participants were categorized into two groups based on their composite score reflecting their general knowledge of HPV (Fig 1). Among all participants, only 19 (5%) demonstrated good knowledge of HPV. There was no significant difference in the percentage of females (5.1%) and males (4.7%) exhibiting good knowledge of HPV. A slightly higher percentage of participants from the University (6.1%) exhibited a good understanding of HPV compared to participants from the College of Education (4.2%). Employed participants showed a higher level of knowledge of HPV compared to unemployed participants. Employment status was found to be significantly associated with participants’ knowledge of HPV (p = .047) (**Table 3**).

**Table 3:** General knowledge of HPV by respondents’ demographic characteristics.

Variables	General Knowledge of HPV		p-value
	Poor N = 360	Good N = 19	
	n (row %)	n (row %)	
Age [mean(sd)]	23.2 (0.2)	23.9 (0.6)	.238
**Gender**			.869
Female	259 (94.9)	14 (5.1)	
Male	101 (95.3)	5 (4.7)	
**Institution**			.412
PLASU	155 (93.9)	10 (6.1)	
FCE	205 (95.8)	9 (4.2)	
**Educational Level**			.309
100L	93 (92.1)	8 (7.9)	
200L	77 (97.5)	2 (2.5)	
300L	149 (96.1)	6 (3.9)	
400L	30 (90.9)	3 (9.1)	
Others	9 (100)	0	
**Marital Status**			.383
Married	39 (97.5)	1 (2.5)	
Single	321 (94.7)	18 (5.3)	
**Religion**			.362
Christianity	348 (95.3)	17 (4.7)	
Islam	8 (88.9)	1 (11.1)	
**Employment**			.047
Yes	111 (91.7)	10 (8.3)	
No	246 (96.5)	9 (3.5)	

Others—Students who are spending extra years on campus

The results of both crude and adjusted odds ratios of having good knowledge of HPV were presented in **Table 4**. When adjusted for other variables, each one-year increase in age was associated with a 21% increase in the odds of having good knowledge of HPV (AOR = 1.21, 95% CI: 1.00–1.47). The odds of having good knowledge of HPV were lower among male participants (AOR = 0.75, 95% CI: 0.22–2.55) compared to female participants. Federal College of Education participants had 1.74 times higher odds (AOR = 1.74, 95% CI: 0.49–6.16) of having good knowledge of HPV than University participants. Furthermore, participants who were employed significantly had good knowledge of HPV compared to those who were unemployed (AOR = 0.32, 95% CI: 0.11–0.88).

**Table 4:** Logistic regression analysis of factors associated with good HPV knowledge.

Variables	Crude OR (95% CI)	p-value	Adjusted OR (95% CI)	p-value
**Age**	1.09 (0.93–1.29)	.285	1.21 (1.00–1.47)	.050
**Gender**		.869		.650
Female	1		1	
Male	0.92 (0.32–2.61)		0.75 (0.22–2.55)	
**Institution**		.414		.389
PLASU	1		1	
FCE	0.68 (0.27–1.72)		1.74 (0.49–6.16)	
**Educational level**		.267		.104
100L	1		1	
200L	0.30 (0.06–1.46)		0.18 (0.03–1.00)	
300L	0.47 (0.16–1.39)		0.20 (0.05–0.84)	
400L	1.16 (0.29–4.66)		0.58 (0.11–3.00)	
Others	1		1	
**Marital status**		.452		.328
Married	1		1	
Single	2.19 (0.28–16.83)		3.11 (0.32–30.21)	
**Religion**		.388		.257
Christian	1		1	
Islam	2.56 (0.30–21.64)		3.77 (0.38–37.37)	
**Employment**		.057		.027
Yes	1		1	
No	0.41 (0.16–1.03)		0.32 (0.11–0.88)	

Others—Students who are spending extra years on campus

## Discussion

This study found that awareness of HPV and its vaccination is low among tertiary institution students in Plateau State, Nigeria. The students’ knowledge of HPV was generally poor. Few students had good knowledge of HPV, and only a small percentage knew that HPV causes cervical cancer and that HPV could be prevented with administration of vaccine.

The low awareness/knowledge of HPV in this study are similar to the findings from a study conducted in Lagos and another study in Kano, where it was found that only 17.7% and 3.7% of female participants, respectively, had good awareness and knowledge of HPV [23, 24].Given that every participant had finished secondary school education, the similarity of our study’s findings to those conducted in Lagos was unexpected. The lack of state-sponsored health education initiatives about HPV infection and vaccination programs could have accounted for the observed low level of awareness of HPV, which is believed to be a problem in the majority of low-and middle-income countries [25, 26]. However, further analysis revealed a significant association between educational level and HPV awareness. This association aligns with findings from studies conducted in developed countries, which also reported a relationship between increased levels of education and HPV awareness [27–29].

The prevention of HPV infections is essential for the control of HPV-related cancers. The study participants had extremely low levels of awareness of the HPV vaccine. Only 8.01% of respondents have gotten the HPV vaccine, indicating that low awareness of the vaccine and its importance has a detrimental effect on vaccine uptake. Compared to a previous studies conducted in Benin City, Nigeria [30], there was a higher level of awareness about the HPV vaccine in Plateau state. In contrast to our study, the findings of other studies conducted in Ethiopia (31.4%) [31], Switzerland (70.7%) [32], and China (69.2%) [33] among students, showed more awareness about the HPV vaccine. Furthermore, the vaccine uptake is significantly lower than the HPV vaccine uptake recorded in high-income countries such as in Germany (67.0%) [29]. It is plausible that the observed variation could be due to the differences in geographical location, government policies, healthcare activities, awareness campaigns, and socioeconomic factors. Lack of accessibility to the vaccine is another issue lowering HPV vaccination rates.

Although the knowledge score of HPV was low in males and females in this study, but the knowledge score were lower in male students relative to female students. It is possible that the poor knowledge in males compared to women might be due to the general belief of associating HPV with cervical cancer than other HPV associated cancers. In addition, the government of Nigeria introduced the funded HPV vaccination among girls with little or no information or investment on vaccinating the boys.

There were significant differences in the perceptions of HPV transmission and preventive techniques, particularly between male and female students. Most respondents agreed on certain modes of transmission such as unprotected intercourse and blood transfusions, but discrepancies were found in other methods. For example, only 48% of female participants and 60% of male participants acknowledged that French kissing is a potential mode of transmission. However, there was a notable difference in the methods to prevent HPV among male and female participants. For example, a higher percentage of female participants believe that using a barrier method during sexual activity could prevent HPV transmission compared to male participants. Conversely, the majority of participants, regardless of their sex, recognized the importance of remaining faithful in a relationship as a preventive measure for HPV infection. The cultural norms surrounding health talks and specialized health efforts that focus on women’s health issues may have influenced female participants, who demonstrated a remarkable knowledge of HPV preventive techniques.

This study has some limitations: the data were collected from a self-administered questionnaires and this could have introduced some reporting bias. It is also likely that social desirability bias might affect the choice of response in a conservative environment like plateau state. Self-report bias and recall bias may have been present because we relied on self-reported data gathered through self-administered questionnaires. The cross-sectional design of this study limits the potentials to draw causality on the findings from the study.

In conclusion, this study highlights the inadequate awareness and knowledge of HPV, its transmission, and preventive measures among students in Plateau State tertiary institutions. Targeted educational programs focusing on diverse educational levels and institution types are recommended to enhance HPV knowledge and promote vaccine uptake. These include workshops, peer education initiatives, and curriculum integration, leveraging online platforms and on-campus events to effectively enhance HPV knowledge and promote vaccine uptake among students in Plateau State tertiary institutions.

Future research should focus on longitudinal studies to assess the sustained impact of educational interventions on HPV knowledge retention and behavioural changes among students in Plateau State tertiary institutions. There is a critical need for advocacy aimed at integrating HPV vaccination into the national immunization programs and school health policies. This advocacy should be informed by regional studies and tailored to the cultural and educational context of Plateau State and similar regions in Nigeria. Furthermore, exploring innovative strategies to overcome barriers to HPV vaccine uptake, such as addressing vaccine hesitancy and accessibility issues, should be a priority for both research and policy efforts. These initiatives are essential for advancing public health outcomes and reducing HPV-related health risks in the state and the country at large.

## Supporting information

S1 Text Quantitative questionnaires. (DOCX)
