# Supplementary material for: Assessment of knowledge of human papillomavirus transmission and prevention among tertiary institution students in the Plateau Central Senatorial District, Nigeria
Source: PLOS Glob Public Health. 2024 Sep 27;4(9):e0003273. doi: 10.1371/journal.pgph.0003273 (PMC11432872; doi:10.1371/journal.pgph.0003273)
Supplement: S1 Text — (DOCX) [file pgph.0003273.s001.docx]

**APPENDIX B**

[
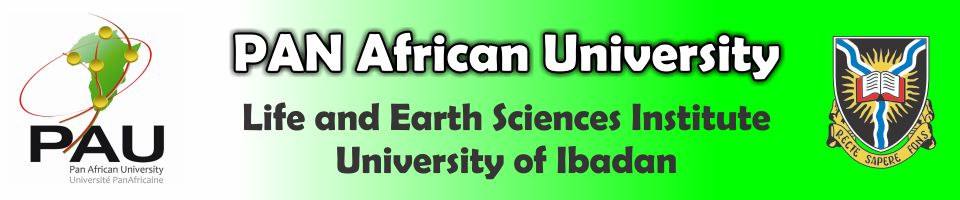
](http://elspau.ui.edu.ng/)**INFORMED CONSENT FORM FOR COLLECTION OF BLOOD, ORAL AND GENITAL SAMPLES.**

IRB Research approval number: UI/EC/21/0028

This approval will elapse on:

**Title of the study: Non-penile Vaginal sexual Behaviour and Associated Health Risks Among Young Adults in Higher Institutions of Central Senatorial District of Plateau State Nigeria.**

I Juliana Rume a PhD Reproductive Biology student of the Department of Obstetrics and Gynaecology University College Hospital University of Ibadan humbly request your kind participation in the above-mentioned ongoing study by responding honestly to questions provided in either IDI, FGDs or questionnaire. I appeal for your kind compliance during sample collection to enable me achieve the objectives of my studies.

**The purpose of the research**

The purpose of this research is to assess the pattern of Non-Penile-Vaginal sex and associated health risk among young adults in tertiary institutions in Plateau state (North central) Nigeria

This study will benefit participants since testing is free and expert recommend annual or more test for sexually active individual within the ages of 25 and below for the infections to be screened for in this study. Results from this research will published as part of my thesis without including any information that could identify you and if you wish to personally know your result of test performed on you, it will be made available to you. will be made available to the institutions and accessed by participants if they so wish.

**Procedure of the research**

A total of 220 students are expected to participate in this research in two institutions giving a total of 440 students to meet up the 385-population sample calculated. Each participant is to participate in either FGD, IDI or cross-sectional studied and present for sample collection afterwards.

**Expected duration of research and of participant(s)’ involvement:**

This research is proposed to last for a year to cover the FGDs, IDI, cross sectional studies and subsequent sample collection.

**Risk(s):** A not too severe risk of pain from needle pricks from blood sample collections and some discomfort during genital and oral self-sample swab collection shall be experienced.

**Confidentiality and privacy in the study:** Your name and identity if you consent to participate in this study will not be in any way revealed during the reporting and discussions of data from this study and strict confidentiality will be maintained.

**Joining and withdrawing from the study:** Participation in the study is voluntary and you will not be forced to join the study. You will not suffer any consequences if you decide not to participate in the study or in the event of your decision to withdraw from the study. Please note that some of the information that has been obtained about you before you chose to withdraw at the point it has been modified or used in reports and publications cannot be removed anymore. However, the researchers promise to make effort in good faith to comply with your wishes as much as is practicable.

**Due inducement**; You will be compensated for cost of transport to and from the research site but you will not be paid any fees for participating in this research. If you suffer any injury as a result of your participation in this research, you will be treated at the institutions clinic.

**Statement of person obtaining informed consent:**

I have fully explained this research to ____________________________________ and have given sufficient information, including about risks and benefits, to make an informed decision.

DATE: _____________________ SIGNATURE: ____________________________

NAME: ______________________________________________

Participants ID number …………………………………………

**Statement of person giving consent:**

I have read the description of the research and have had it translated into language I understand. I have also discussed with the doctor to my satisfaction. I understand that my participation is voluntary. I know enough about the purpose, methods, risks and benefits of the research study to judge that I want to take part in it. I understand that I may freely stop being part of this study at any time. I have received a copy of this consent form and additional information sheet to keep for myself.

DATE: ___________________ SIGNATURE: _________________________________

NAME: _____________________________________________

Thank you.

Date of interview

Time of interview

INTERVIEW GUIDE FOR FGD AND IDI.

I wish to start by asking you the following questions. While growing up in your different community’s primary to secondary school, I will like to know,

1. What do you understand by the word sexual intercourse?

Probe for sexual intercourse between opposite sex and between same sex LGBTQ.

1. Probe for general meaning: who are involved (between men and women men and men, and between women and women)
2. Probe for specific meaning of sexual acts.
3. What are the different methods of sexual activity that you know?
4. Probe for penetration between (penis and vagina, penis and mouth, penis and anus); (hand and vagina, hand and anus); (use of sharp or toy and vaginal and use of objects or toy and anus).
5. Probe for use of non-penetrative materials (masturbation-touching yourself to stimulate arousal and mutual masturbation- touching each other with your partner to stimulate arousal) and use of other erotic materials e.g music, film, picture etc.
6. What are the motivations for people to engage in these different types of activities, financial (transactional sex), peer pressure, Satisfy curiosity, biological needs, Marriage purpose, Voluntary, partner pressure?
7. What other special terms are used by people to refer to those sexual behaviours apart from penile vaginal sex? (Oral sex, masturbation, anal sex, use of sex toys/objects?
8. How are the people who engaged in these other sexual behaviours viewed within this community?
9. Are there local terminologies or names people use in reference to these names that are only know or used within these age groups?
10. Why do people use those special terms?
11. What reasons do people give for engaging in these other forms of sexual behaviours.
12. Are you aware of any consequences or side effects to those sexual practices as Oral sex, masturbation, anal sex, and use of sex toys/objects?

**RESPONDENTS SHOULD BE WITHING THE AGES OF 18-30**

**SOCIO-DEMOGRAPHIC DATA.**

**Please tick the right option/ fill in the space with the correct answer.**

1. Age ………………………………
2. Gender: Female
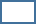
 Male
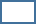

3. Name of Institution

Plateau State University Bokkos (PLASU)
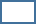


Federal College of Education Pankshin (FCE)
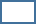


1. Education level

NCE 1
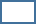
 100level
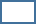


NCE 2
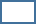
 200level
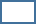


NCE 3
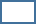
 300level
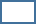


Other
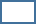
, 400level
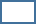


specify………………………………………………………….

1. Marital status

Married
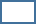


Single
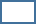


Separated
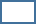


Widowed
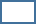


Co-habiting
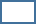


1. State of origin………………………………………………………
2. Religion: Christianity
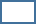
Islam
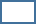
Traditional worshiper
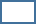
others specify……………...
3. Tribe, Hausa
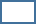
Igbo
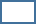
 Yoruba
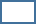
 others(specify)…………………………………….
4. Do you have any form of work/Job doing? Yes
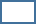
 No
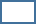

5. If your answer to question 9 is yes please chose the king of work you do.

Selling cloths/materials,
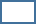
 selling food stuff,
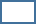
 serving in a restaurant,
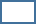
 commercial sex worker,
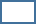
 tailoring,
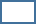
hair dressing,
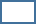
 carpentry,
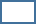
 mechanic,
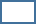
 government employee,
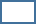
 Sales person,
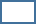


SECTION B

FROM QUESTION 11 TO 16 SELECT THE OPTION THAT BEST DEFINES THE NPVS IN QEUESTION

1. Oral sex means or is

|  | **Oral sex meaning** | **YES** | **NO** |
| --- | --- | --- | --- |
| 11.1 | sexual contact between the mouth and the genitals | **\|______\|** | **\|______\|** |
| 11.2 | sexual contact between the mouth and the anus | **\|______\|** | **\|______\|** |
| 11.3 | sexual contact between the mouth and any body part | **\|______\|** | **\|______\|** |
| 11.4 | sexual contact between the mouth and other objects | **\|______\|** | **\|______\|** |

1. Anal sex means or is

|  | **Aral sex meaning** | **YES** | **NO** |
| --- | --- | --- | --- |
| 12.1 | sexual contact between the mouth and the genitals | **\|______\|** | **\|______\|** |
| 12.2 | sexual contact between the mouth and the anus | **\|______\|** | **\|______\|** |
| 12.3 | sexual contact between the mouth any body part | **\|______\|** | **\|______\|** |
| 12.4 | sexual contact between the mouth and other objects | **\|______\|** | **\|______\|** |

1. Mutual masturbation means or is

|  | **Mutual masturbation means** | **YES** | **NO** |
| --- | --- | --- | --- |
| 13.1 | when two partners use their hands or toys to stimulate each other's genitals for sexual pleasure | **\|______\|** | **\|______\|** |
| 13.2 | when two partners use their genitals to stimulate each other's genitals for sexual pleasure | **\|______\|** | **\|______\|** |
| 13.3 | when two partners use their mouth to stimulate each other's genitals for sexual pleasure | **\|______\|** | **\|______\|** |
| 13.4 | when two partners use objects to stimulate each other's genitals for sexual pleasure | **\|______\|** | **\|______\|** |

1. Self-masturbation means or is

|  | **Self-masturbation means** | **YES** | **NO** |
| --- | --- | --- | --- |
| 14.1 | Touching and rubbing parts of your body for sexual pleasure | **\|______\|** | **\|______\|** |
| 14.2 | Touching and rubbing parts of someone’s body for sexual pleasure | **\|______\|** | **\|______\|** |
| 14.3 | Touching and rubbing parts of animal body for sexual pleasure | **\|______\|** | **\|______\|** |
| 14.4 | Touching and rubbing objects for sexual pleasure | **\|______\|** | **\|______\|** |

1. Sex toys means or is

|  | **Sex toys means** | **YES** | **NO** |
| --- | --- | --- | --- |
| 15.1 | An object or device used for sexual stimulation or to enhance sexual pleasure. | **\|______\|** | **\|______\|** |
| 15.2 | An animal used for sexual stimulation or to enhance sexual pleasure. | **\|______\|** | **\|______\|** |
| 15.3 | An object used for sexual stimulation or to enhance sexual pleasure. | **\|______\|** | **\|______\|** |
| 15.4 | A device used for sexual stimulation or to enhance sexual pleasure. | **\|______\|** | **\|______\|** |

1. French Kiss means or is

|  | **French Kiss means** | **YES** | **NO** |
| --- | --- | --- | --- |
| 16.1 | A kiss with contact between tongues | **\|______\|** | **\|______\|** |
| 16.2 | A kiss with NO contact between tongues | **\|______\|** | **\|______\|** |
| 16.3 | A kiss with contact between tongues and body parts | **\|______\|** | **\|______\|** |
| 16.4 | A kiss with contact between tongues and objects | **\|______\|** | **\|______\|** |

1. Which of these sexual acts are mostly practiced among young adults in school?

|  |  | **YES** | **NO** |
| --- | --- | --- | --- |
| 17.1 | Mutual masturbation, | **\|______\|** | **\|______\|** |
| 17.2 | Self-masturbation | **\|______\|** | **\|______\|** |
| 17.3 | Use of sex toys/object | **\|______\|** | **\|______\|** |
| 17.4 | French kissing | **\|______\|** | **\|______\|** |
| 17.5 | Penile vaginal sex | **\|______\|** | **\|______\|** |

Others
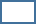
 specify others above………………………………………………………

1. Have you ever felt sexually attracted to some one of same sex as you? Yes
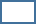
 No
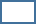

2. Have you ever had sexual contact with some one of the same sex as you? Yes
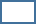
 No
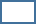

3. Do you think Sexually Transmitted infections can be transmit through Anal sex Yes
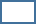
 No
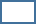

4. Do you think Sexually Transmitted Iinfections can be transmit through Oral sex Yes
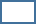
 No
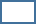

5. Do you think any infection at all can be transmit through self masturbation Yes
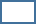
 No
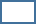

6. Do you think Sexually Transmitted Iinfections can be transmit through mutual masturbation Yes
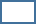
 No
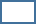

7. Do you think Sexually Transmitted infections can be transmit through use and shareing of sex toys/object Yes
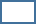
 No
8. Age of your first Non-Penile vaginal sex ………………

At what age did you first practice sex that is between your sex organ and other things apart from the

1. Reasons people engage in non-Penile vaginal sex

| s/no | Reason for practicing NPVS. | Yes | No |
| --- | --- | --- | --- |
|  | Biological needs eg, sex hormone and sexual effects of drug |  |  |
|  | Financial needs, (money or material things) |  |  |
|  | Satisfy curiosity (just wanting to know what it is like) |  |  |
|  | Voluntary |  |  |
|  | Partner pressure |  |  |
|  | Peer pressure |  |  |
|  | Marriage purpose |  |  |
|  | Prevent disease and Pregnancy |  |  |

Others …………………………………………………………………………………...

1. How old were you when you first practice penile vaginal sex? …………………………
2. Number of sex partners in the past 3 months? or How many people have you had sexual contact with within the past 3 months .........................................?
3. How old were you when you first practiced oral sex…………………………?
4. How old were you when you first practiced anal sex…………………………...?
5. How old were you when you first practiced self-masturbation…………………?
6. How old were you when you first practiced mutual masturbation………………?
7. How old were you when you first practiced use of sex toy or other objects………………?
8. Condom was used during last anal sex. Or did you use condoms the last time you had anal sex. Yes
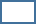
 No
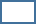

9. Have you ever heard of Dental dam? yes
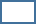
 No
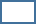

10. Dental dam was used during/ did you use dental dam during the last oral sexual? Yes
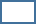
 No
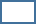

11. Do you drink alcohol Yes
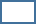
 No
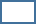

12. How often do you drink? regularly
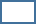
 occasionally
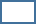

13. Do you smoke cigarette Yes
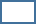
 No
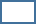

14. Do you take any of these substances: Marijuana (weed)
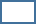
 Codeine
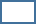
Glue/Gum
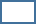
Tramadol
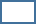
Cocaine
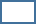
Heroine
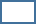
others
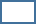
 specify…………………………………?
15. Do you have an android phone? Yes
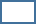
 No
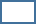

16. Do you have Access to internet facility Yes
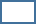
 No
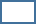

17. How many hours a day are you online. ………………………….
18. Do you attend night clubs/parties? yes
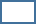
 No
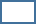

19. How often do you attend clubs/parties? regular
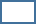
 occationally
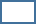

20. Ever had a Sexually Transmitted Infection (STI)? yes
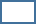
 No
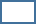

21. Specify the type of STI you ever had please……………………….
22. Were you treated of the STI? Yes
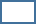
No
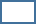
 Currently been managed
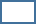

23. Was your sexual partner treated of the STI? Yes
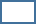
No
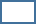
 Currently been managed
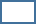

24. Where did you treat or managing the STI? Hospital
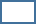
 Traditional medicine
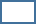

25. Have you ever heard of Human Immunodeficiency Virus **(**HIV)? yes
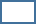
 No
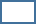

26. Do you know your HIV status? Yes
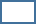
 No
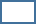

27. In which of the following acts can HIV be transmitted. (Tick all options you agree with)

| a | French kissing |  |
| --- | --- | --- |
| b | Blood transfusion |  |
| c | Sex (oral, anal or vaginal) without dental dams or condoms |  |
| d | Sharing sharp objects |  |
| e | Sharing utensils |  |
| f | Mosquito bite |  |
| g | Sleeping together |  |

1. Which of these ways can HIV be prevented? (Tick all options you agree with)

| a | Not sharing utensil |  |
| --- | --- | --- |
| b | Proper blood screening before transfusion |  |
| c | Use of dental dams or condom during sex |  |
| d | Avoid Sharing sharp objects |  |
| e | Avoid Sharing utensils |  |
| f | Avoiding Mosquito bite |  |
| g | Avoid sleeping with others. |  |
| h | Having one sex partner and remaining faithful |  |

1. Which of these NPVS can transmit HIV? Oral sex
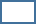
, Kissing
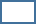
, mutual masturbation
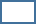
, anal sex
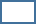
 None of the above
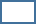

2. Ever heard of, Hepatitis B Virus, (HBV)? Yes
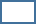
 No
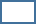

3. Do you know your Hepatitis B Virus status, yes
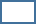
 No
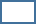

4. In which of the following ways can Hepatitis B Virus be transmitted. Tick all options you agree with

| a | French kissing |  |
| --- | --- | --- |
| b | Blood transfusion |  |
| c | Sex (oral, anal or vaginal) without dental dams or condoms |  |
| d | Sharing sharp objects |  |
| e | Sharing utensils |  |
| f | Mosquito bite |  |
| g | Sleeping together |  |

1. How do you think Hepatitis B Virus can be prevented? Tick all options you agree with

| a | Not sharing utensil |  |
| --- | --- | --- |
| b | Proper blood screening before transfusion |  |
| c | Use of dental dams or condom during sex |  |
| d | Avoid Sharing sharp objects |  |
| e | Avoid Sharing utensils |  |
| f | Avoiding Mosquito bite |  |
| g | Avoid sleeping with others. |  |
| h | Having one sex partner and remaining faithful |  |
| i | Getting vaccinated |  |

1. Are you aware it has a vaccine? yes No
2. Have you been vaccinated? yes No
3. Which of these NPVS act can transmit Hepatitis B Virus? Oral sex, Kissing, mutual masturbation, anal sex None of the above
4. Ever heard of Chlamydia Trachomatis (CT), yes No
5. Ever been tested for Chlamydia Trachomatis (CT), yes No Not sure
6. How do you think Chlamydia Trachomatis (CT), can be transmitted?

| a | French kissing |  |
| --- | --- | --- |
| b | Blood transfusion |  |
| c | Sex (oral, anal or vaginal) without dental dams or condoms |  |
| d | Sharing sharp objects |  |
| e | Sharing utensils |  |
| f | Mosquito bite |  |
| g | Sleeping together |  |

1. How do you think Chlamydia Trachomatis CT can be prevented?

| a | Not sharing utensil |  |
| --- | --- | --- |
| b | Proper blood screening before transfusion |  |
| c | Use of dental dams or condom during sex |  |
| d | Avoid Sharing sharp objects |  |
| e | Avoid Sharing utensils |  |
| f | Avoiding Mosquito bite |  |
| g | Avoid sleeping with others. |  |
| h | Having one sex partner and remaining faithful |  |

1. Which of these NPVS do you think can transmit Chlamydia Trachomatis? Oral sex, Kissing, mutual masturbation, anal sex None of the above
2. Do you think Chlamydia Trachomatis can cause infertility? yes No
3. Ever heard of Human Papilloma Virus (HPV)? yes No
4. Do you know your status, yes No
5. How do you think Human Papilloma Virus can be transmitted?

| a | French kissing |  |
| --- | --- | --- |
| b | Blood transfusion |  |
| c | Sex (oral, anal or vaginal) without dental dams or condoms |  |
| d | Sharing sharp objects |  |
| e | Sharing utensils |  |
| f | Mosquito bite |  |
| g | Sleeping together |  |

1. How do you think it can be prevented? (Tick all options you agree with)

| a | Not sharing utensil |  |
| --- | --- | --- |
| b | Proper blood screening before transfusion |  |
| c | Use of dental dams or condom during sex |  |
| d | Avoid Sharing sharp objects |  |
| e | Avoid Sharing utensils |  |
| f | Avoiding Mosquito bite |  |
| g | Avoid sleeping with others. |  |
| h | Having one sex partner and remaining faithful |  |
| i | Getting vaccinated |  |

1. Are you aware it has a vaccine? yes No
2. Have you been vaccinated? yes No
3. Are you aware it causes cancer? yes No
4. Which of these NPVS do you think can transmit HBV? Oral sex, Kissing, mutual masturbation, anal sex None of the above
